# Activity of alkaloids from *Aspidosperma nitidum* against *Leishmania (Leishmania) amazonensis*

**DOI:** 10.1038/s41598-022-12396-x

**Published:** 2022-05-23

**Authors:** Andreza do Socorro Silva da Veiga, Fernando Tobias Silveira, Edilene Oliveira da Silva, José Antônio Picanço Diniz Júnior, Sanderson Corrêa Araújo, Marliane Batista Campos, Andrey Moacir do Rosário Marinho, Geraldo Célio Brandão, Valdicley Vieira Vale, Sandro Percário, Maria Fâni Dolabela

**Affiliations:** 1 Postgraduate Program in Pharmaceutical Innovation, Institute of Health Sciences, Federal University of Pará, Belém, PA Brazil; 2 Institute Evandro Chagas, Ananindeua, PA Brazil; 3 Postgraduate Program in Biology of Infectious and Parasitic Agents, Institute of Biological Sciences, Federal University of Pará, Belém, PA Brazil; 4 Postgraduate Program in Biodiversity and Biotechnology of the BIONORTE Network, Institute of Biological Sciences, Federal University of Pará, Belém, PA Brazil; 5 School of Pharmacy, Federal University of Ouro Preto, Ouro Preto, MG Brazil; 6 Oxidative Stress Research Laboratory, Institute of Biological Sciences, Federal University of Pará, Belém, PA Brazil

**Keywords:** Biological techniques, Microscopy, Scanning probe microscopy, Transmission electron microscopy, Natural products, Parasitology

## Abstract

This study evaluated the morphological changes caused by fractions and subfractions, obtained from barks of *Aspidosperna nitidum*, against *L*. (*L*.) *amazonensis* promastigotes. The ethanolic extract (EE) obtained through the maceration of trunk barks was subjected to an acid–base partition, resulting the neutral (FN) and the alkaloid (FA) fractions, and fractionation under reflux, yielded hexane (FrHEX), dichloromethane (FrDCL), ethyl acetate (FrACoET), and methanol (FrMEOH) fractions. The FA was fractionated and three subfractions (SF5-6, SF8, and SF9) were obtained and analyzed by HPLC–DAD and ^1^H NMR. The antipromastigote activity of all samples was evaluated by MTT, after that, scanning electron microscopy (SEM) and transmission electron microscopy (TEM) for the active fractions were performed. Chromatographic analyzes suggest the presence of alkaloids in EE, FN, FA, and FrDCL. The fractionation of FA led to the isolation of the indole alkaloid dihydrocorynantheol (SF8 fractions). The SF5-6, dihydrocorynantheol and SF-9 samples were active against promastigotes, while FrDCL was moderately active. The SEM analysis revealed cell rounding and changes in the flagellum of the parasites. In the TEM analysis, the treated promastigotes showed changes in flagellar pocket and kinetoplast, and presence of lipid inclusions. These results suggest that alkaloids isolated from *A. nitidum* are promising as leishmanicidal.

## Introduction

Leishmaniasis is caused by protozoa from the genus *Leishmania*, being endemic in 98 different countries with approximately 350 million people at risk of disease transmission^1^, with 94% of new cases arising only in seven of these countries (Brazil, Ethiopia, India, Kenya, Somalia, South Sudan and Sudan)^1^.

The treatment is carried out with pentavalent antimonials (Sb5 + ; N-methyl glucamine-Glucantime® antimoniate and sodium stibogluconate-Pentostan®) and amphotericin B, these are high-cost chemotherapies, used parenterally and usually require a long administration period^2^. Several adverse reactions and toxic effects have been associated with these drugs^3,4^ and some are dose- and time-dependent^5^. In addition, therapeutic failure has been reported, as well as cases of disease recurrence^6^ and parasitic resistance^7^, associated with an intracellular decrease in drug concentration, due to overexpression of ABC carriers^8,9^.

In this context, it is urgent to search for therapeutic alternatives, and compounds isolated from medicinal plants can be promising^10^. Among several possibilities, there are the alkaloids whose leishmanicidal activity has been described in some studies^11,12^. However, several plant species that present alkaloids have not been studied yet, such as *Aspidosperma nitidum* (Apocynacea family). Therefore, the present study evaluated whether the fractionation of the ethanolic extract (EE) obtained from trunk barks of *A. nitidum* influenced leishmanicidal activity. The study also evaluated the morphological changes in the parasite caused by the active isolated alkaloids.

## Results

### Phytochemical studies

**Figure 1:**
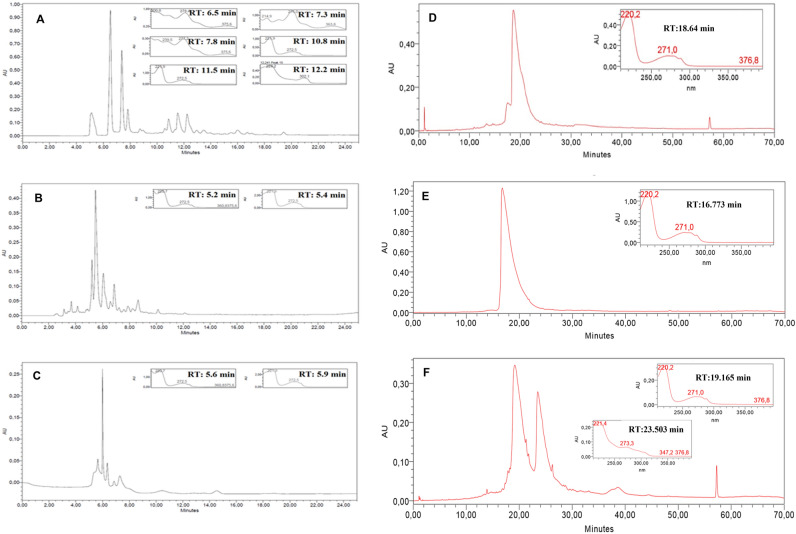
Chromatographic profile and UV spectra of EE, FN, FA and SF5-6, SF8 and SF9 subfractions obtained from *A. nitidum*. Condition: Column RP-18 (45 × 250 mm, 5 µm), λ 220–400 nm, flow = 1 mL/min, temperature > 26 °C. Mobile phase: t = 15 min: 70–30% of eluent B, t = 20 min: 60–40% of eluent B, t = 25 min: 50–100% of eluent B. (**A**)- EE; (**B**)- FA; (**C**)- FN; (**D**)- SF5-6; (**E**)- SF8, (**F**)- SF9.

The yields of EE and fractions are described in Table 1. The analysis in HPLC–DAD detected the presence of alkaloids in the EE (Retention Time (RT) = 6.5 min., Maximum Wave-Length (λmax) 200.8; 278.4 and 375.6 nm; RT = 7.3 min., λmax 214.9, 276.0 and 363.8 nm; RT = 7.8 min., λmax 239.5, 277.2 and 375.6 nm; RT = 10.8 min., λmax 221.8 and 272.5 nm; RT = 11.5 min. , λmax 221.9 and 272.5 nm; RT = 12.2 min., λmax 224.2 and 302.1 nm; Fig. 1A), the alkaloid fraction (FA; RT = 5.2 and 5.4 min; λmax 220.7 and 272.5 nm; Fig. 1B), and in the neutral fraction (FN; RT = 5.6 and 5.9 min .; λmax 220.7 and 272.5 nm; Fig. 1C), and suggested that subfractions SF 5–6 (RT = 18.64 min.; λ max. 220.2 and 271.0 nm; Fig. 1D), SF8 (RT = 16.773 min.; λmax 220.2 and 271.0 nm; Fig. 1E), and SF9 (RT = 19.165 min.; λmax. 220.2 and 271.0 nm and RT 23.503 min.; λmax 221.4 and 273.3 nm; Fig. 1F) must be alkaloids.

**Table 1:** Yields of ethanolic extract and its fractions obtained by acid–base partition or reflux extraction.

Sample	Yields (%)
EE^a^	5.1
FA^b^	29.25
FN^c^	21.8
FrHEX^d^	2.4
FrDCL^e^	8.37
FrACOET^f^	18.6
FrMeOH^g^	38.4

^a^EE:ethanolic extract; ^b^FA: alkaloids fraction; ^c^FN: neutral fraction; ^d^FrHEX: hexane fraction; ^e^FrDCL: dichloromethane fraction; ^f^FrACOET- ethyl acetate fraction; ^g^FrMEOH- methanolic fraction.

**Figure 2:**
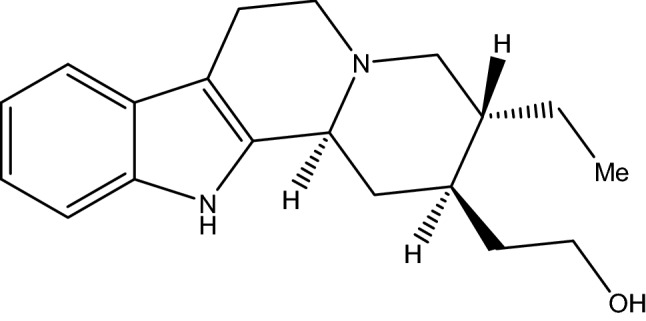
Structure and numbering system of SF8 subfraction (dihydrocorinantheol) based on its biogenesis.

^**1**^**H NMR SF8** (400 MHz, CD3OD); δ 7.40 (d, Ar-H, H-9), 7.28 (d, Ar-H, H-12), 7.05 (Ar-H, H-11), 6.99 (Ar-H, H-10), 3.68 (m, H-17), 3.49, 3,24 (5H), 3.20 (H-21), 3.03 (6H), 2.80 (6H), 2.77 (d, 5H), 2.52 (d, H-14), 2.30, 2,15 (s), 2.01 (H-16), 1.98 (m), 1.75 (m, H-19’), 1.53 (m), 1.50, 1.35 (m, H-16’), 1.30, 0.94 -1.25 (t, H-19).

The analysis of the ^1^H NMR spectrum of the SF5-6 subfraction, showed signs of hydrogen with displacements in δH 7.47, 7.35, 7.07, and 7.19 that correspond to aromatic hydrogens. Meanwhile, SF8 ^1^H NMR spectrum (see signs below) demonstrated the presence of three signals with chemical shifts in δH 7.40 for H9, δH 7.28 for H12, δH 7.05 for H11 (Fig. 2). The ^1^H NMR of the SI9 subfraction showed four δH 7.49, 7.37, 7.15, and 7.06 signals that were attributed to aromatic ring hydrogens.

### Anti-promastigote activity and morphological analysis

When considering the antipromastigote activity, Table 2 shows the 50% inhibitory concentrations (IC_50_%) and that only samples SF5-6, SF-9 and dihydrocorynantheol were active, while FrDCL was moderately active.

**Table 2:** Antipromastigote activities of *Aspidosperma nitidum*.

Sample	*L. (L.) amazonensis*	
	IC_50_^a^ (µg/mL) ± SD^b (1)^	Activity
SF 5–6	100.3 ± 1.8	Active*
Dihydrocorynantheol	38.4 ± 3.1	Active
SF9	62.4 ± 1.9	Active
FrDCL^c^	105.7 ± 1.1	Moderate
Amphotericin B	0.06 ± 0.002	Active

^a^IC_50_: 50% inhibitory concentration; ^b^SD: Standard deviation; ^c^FrDCL: dichloromethane fraction; (1) 95% confidence interval.

**Figure 3:**
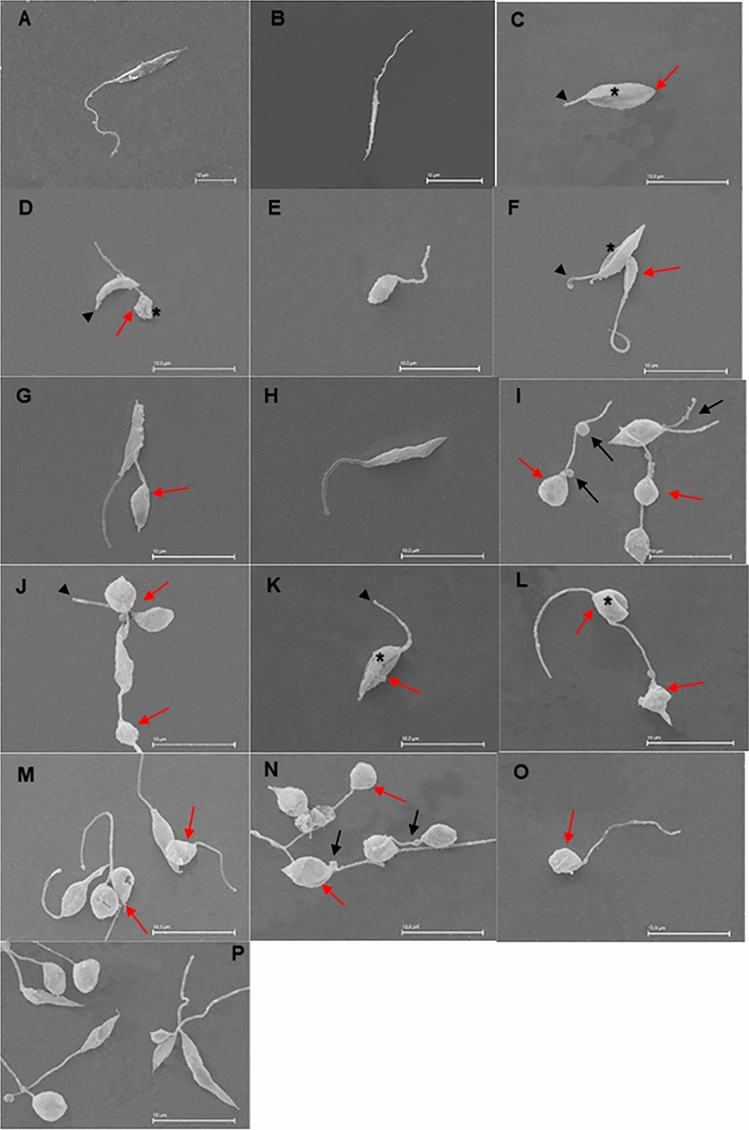
Ultrastructural changes in *L. (L) amazonensis* promastigotes treated with amphotericin B, FrDCL, SF5-6, SF8 and SF9 obtained from *Aspidosperma nitidum* bark. (**A**) Negative control; (**B**) Solvent control; (**C**, **D** and **E**) amphotericin B: 0.1953 µg/mL, 0.09765 µg/mL and 0.048825 µg/mL, respectively; (**F**, **G** and **H**) FrDCL: 50 µg/mL, 25 µg/mL and 12.5 µg/mL, respectively; (**I** and **J**) SF5-6: 50 µg/mL and 25 µg/mL, respectively; (**K**, **L** and** M**) dihydrocorynantheol: 25 µg/mL, 12.5 µg/mL and 6.25 µg/mL, respectively; (**N**, **O** and **P**) SF9 50 µg/mL, 25 µg/mL and 12.5 µg/mL, respectively. The arrowhead indicates a parasite with a short flagellum, a black arrow indicates protuberances in the flagellum and/or a double flagellum, the red arrow indicates a change in cell shape and the asterisk, septation of the cell body. Bars: 10 µm.

In the scanning electron microscopy (SEM) analysis, promastigotes treated with the dichloromethane fraction of EE (FrDCL) displayed alterations in a concentration-dependent fashion. We observed cell rounding, membrane septation and shortening of the flagellum size in parasites treated with 50 µg/mL of FrDCL (Fig. 3F). At a concentration of 25 µg/mL, promastigotes still presented rounded-shape cells (Fig. 3G), and in parasites treated with 12.5 µg/mL, there were no significant changes (Fig. 3H).

Similarly, in parasites treated with SF5-6, SF8, and SF9, as analyzed by SEM, changes were concentration-dependent. For the parasites treated with 50 µg/mL of SF5-6, a short flagellum was observed in most of them, as well as a double flagellum and small protuberances along the flagellum in some of the promastigotes (Fig. 3I), and cell rounding. At the lowest concentration (25 µg/mL), the features displayed were flagellum shortening and cell rounding (Fig. 3J).

The parasites treated with 25 µg/mL of dihydrocorynantheol presented a change in shape of the cell body, which was septate, and flagellum shortening (Fig. 3K). Promastigotes treated with 12.5 µg/mL displayed rounded shape, some displaying cell body septation, but no promastigotes with a short flagellum were observed (Fig. 3L). For the parasites treated with 6.25 µg/mL of dihydrocorynantheol, only rounding cells were observed (Fig. 3M).

Moreover, for the parasites treated with 50 µg/mL of SF9, a round shape and protuberances were observed along the flagellum (Fig. 3N), while at 25 µg/mL there were cells with rounded shape (Fig. 3O), and at 12.5 µg/mL most parasites presented an elongated cell body, a long flagellum, and no protuberances (Fig. 3P).

No significant changes were observed for the negative control group (NC; Fig. 3A), or for the solvent control group (CSOL; Fig. 3B). The main changes caused by the treatment with Amphotericin B were changes in flagellum size, loss of elongated shape, septation of the cell body (0.1953 µg/mL; Fig. 3C). For the promastigotes treated with 0.09765 µg/mL, the same changes were observed, except for the changes in flagellum size (Fig. 3D). The promastigotes treated with 0.048825 µg/mL presented more elongated cells, but septation or flagellum shortening was not observed (Fig. 3E).

**Figure 4:**
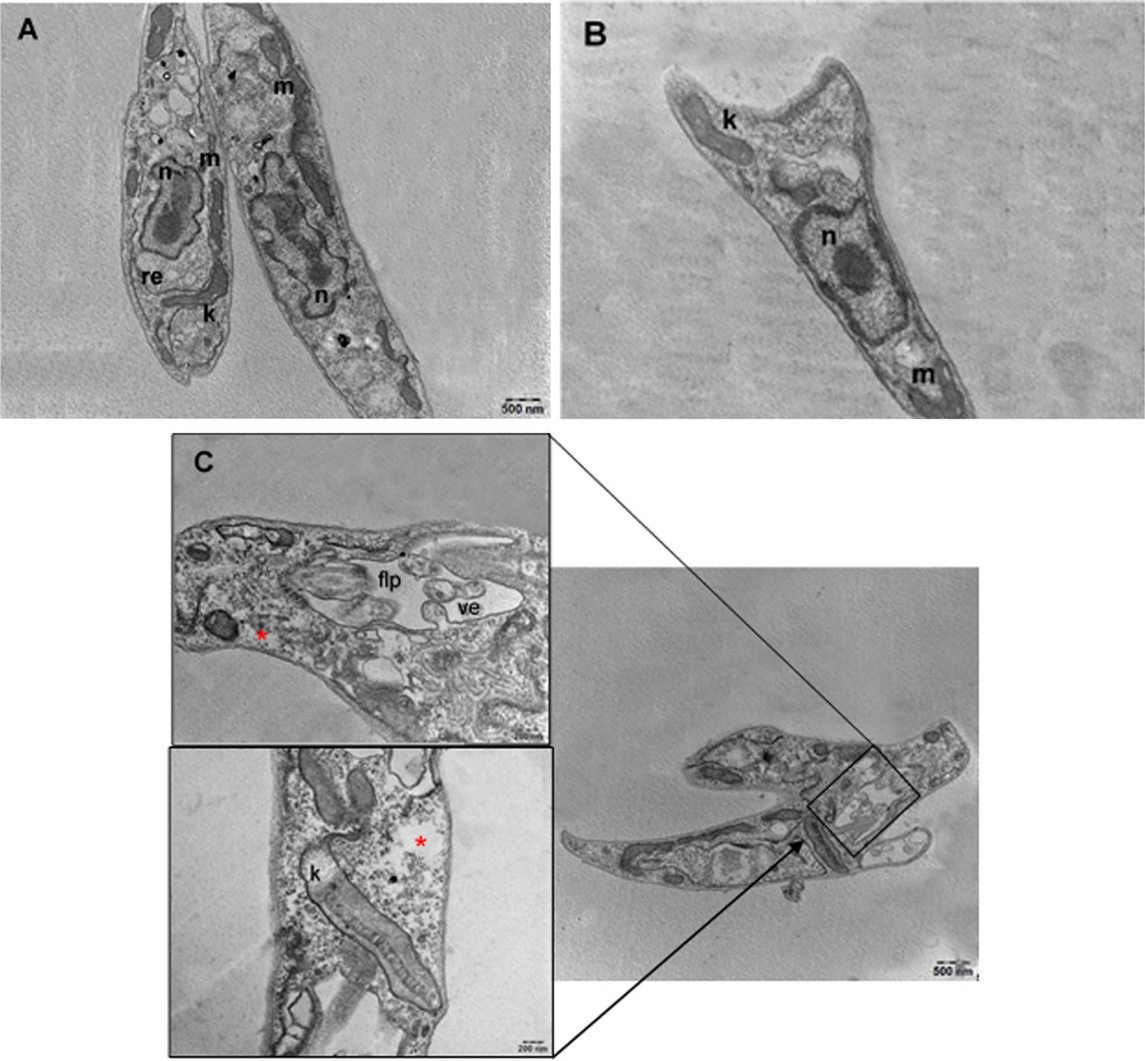

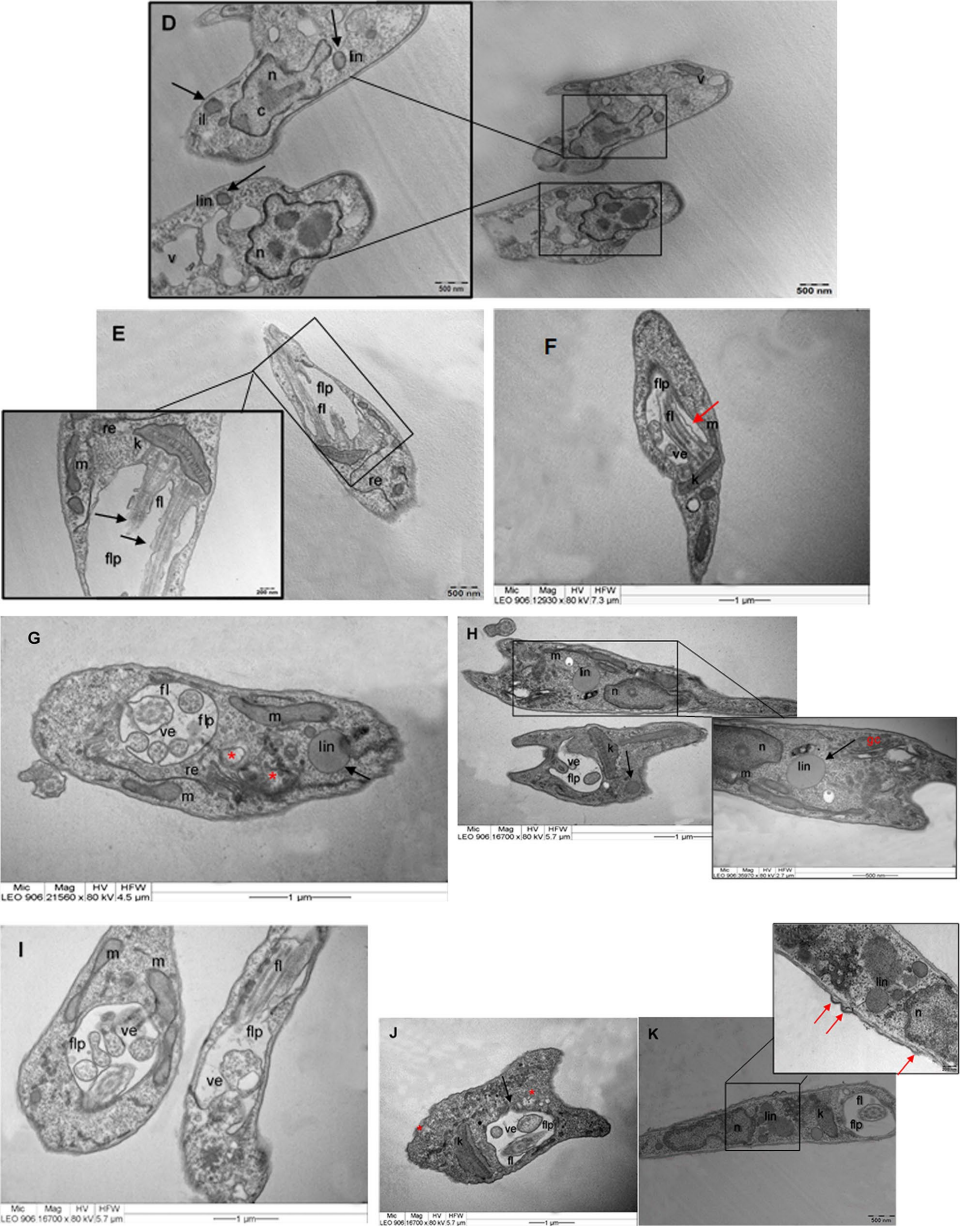
Ultrastructural aspects of *L. (L). amazonensis* promastigotes untreated and treated with samples for 72 h. (**A**) Negative control; (**B**) Solvent control; (**C**) Promastigotes treated with 50 µg/mL of FrDCL with retraction of the cell body, dilated flagellar pocket with vesicles inside it and presence of valcuoles in the cytoplasm (red asterisk); (**D**) Promastigotes, treated with 25 µg/mL of FrDCL with rounded cell, lipid inclusion (black arrow), nucleus apparently with fragmented chromatin (red arrow), and vacuoles in the cytoplasm; **(E)** Promastigotes treated with 12.5 µg/mL of FrDCL with an enlarged flagellar pocket, and alteration in the flagellar membrane (black arrow); (**F**) Promastigotes treated with 50 µg/mL of SF5-6 presenting dilated flagellar pocket, with vesicles inside, and altered flagellar membrane (red arrow); (**G**) Promastigotes treated with 25 µg/mL of dihydrocorynantheol, showed retraction of the cell body, dilated flagellar pocket, with vesicles inside and presence of lipid inclusion (black arrow) and vacuoles in the cytoplasm (asterisk); (**H**) Promastigotes treated with 12.5 µg/mL of dihydrocorynantheol, with changes in the flagellar pocket, with vesicles inside, lipid inclusion (black arrow), dilation of the Golgi complex cisterns. (**I**) Promastigotes treated with 6.26 µg/mL of dihydrocorynantheol, with a change in the flagellar pocket, with vesicles inside; (**J**) Promastigotes treated with 50 µg/mL of SF9 with retraction of the cell body, alteration in the flagellar pocket, that has vesicles inside it, vacuoles in the cytoplasm (asterisk), and dilated kinetoplast; (**K**) Promastigotes treated with 25 µg/mL of SF9 with alteration in the shape of the flagellar pocket,, dilated kinetoplast, presence of lipid inclusions and projections of the cytoplasmic membrane (red arrows). (flp) flagellar pocket; (c) chromatin; (gc) Golgi complex; (k) kinetoplast; (fl) flagellum; (lin) lipid inclusions; (m) mitochondria; (n) nucleus; (er) endoplasmic reticulum; (v) vacuoles; (ve) vesicles.

Additionally, other groups of promastigotes were treated with the same samples (FrDCL, SF5-6, SF8, and SF9) and the morphological changes were evaluated by transmission electron microscopy (TEM). For the NC, no subcellular changes were observed (Fig. 4A), as well as in the CSOL (Fig. 4B). Parasites treated with FrDCL (50 µg/mL) presented important cellular alterations, such as complete loss of normal morphology, retraction of the cell body, cytoplasmic disorganization, dilated flagellar pocket with evagination of its membrane for possible formation of vesicles, and cytoplasmic vacuolization (Fig. 4C). The following changes were observed for the parasites treated with 25 µg/mL of FrDCL: rounding cells, lipid accumulation, apparently fragmented chromatin in the nucleus, and an increase in the number of vacuoles in the cytoplasm (Fig. 4D). The treatment of promastigotes with 12.5 µg/mL induced dilatation of the flagellar pocket and alteration in the flagellar membrane (Fig. 4E).

In promastigotes treated with 50 µg/mL of SF5-6, an enlarged flagellar pocket with evagination of its membrane for possible formation of vesicles and alteration of the flagellar membrane were observed (Fig. 4F).

Treatment of promastigotes with 25 µg/mL of dihydrocorynantheol induced cytoplasmic disorganization, cell body retraction, complete loss of normal flagellar pocket morphology with vesicles which were probably results of the evagination of the flagellar pocket membrane, and presence of material with a granular aspect inside these vesicles. In addition to these changes, lipid inclusions and vacuoles were observed in the cytoplasm (Fig. 4G). The treatment of promastigotes with 12.5 µg/mL promoted the appearance of lipid inclusions, changes in the flagellar pocket membrane, which presented vesicles with granular material and were probably the result of this evagination, and dilation of cisterns of the Golgi complex (Fig. 4H). For the promastigotes treated with 6.25 µg/mL, there was also a change in the shape of the flagellar pocket and vesicles within presented granular material (Fig. 4I).

For the promastigotes treated with SF9 at 50 µg/mL, we observed retraction of the cell body, alteration in the flagellar pocket morphology, which contained vesicles with granular material inside, kinetoplast dilation, and the presence of small vacuoles in the cytoplasm (Fig. 4J). Promastigotes treated with 25 µg/mL displayed changes in the flagellar pocket shape, dilated kinetoplast, presence of lipid inclusions, and small projections of the parasite's cytoplasmic membrane (Fig. 4K).

## Discussion

To date, no studies that tested the antileishmanial activity of dihydrocorynantheol were found, and this study is the first to test the activity of this indole alkaloid against the parasite of the genus *Leishmania*. Notwitstanding, previous studies isolated different compounds from *A. nitidum:* 10-methoxy-dihydrocorynantheol, corynantheol^13^, harman carboxylic acid^14^, 3- methyl-harman-carboxylic acid, dihydrocorynantheol, dehydrositsiriquine^15^, and braznitidumine^16^. However, none of these alkaloids isolated from *A. nitidum,* and which have a structure similar to dihydrocorynantheol, were tested against species of the genus *Leishmania,* and only one close study was identified, in which the author tested the alkaloid braznitidumine, isolated from the methanolic extract of *A. nitidum,* against the protozoan *Plasmodium falciparum*, but is was inactive^17^. In the present study, dihydrocorynantheol (SF8) was isolated, identified, and tested against *L*. (*L*.) *amazonensis*, and preliminary analyzes suggest that the other fractions (SF5-6 and SF9) are indole alkaloids, but it was not possible to determine their structure.

After analysis in HPLC–DAD, we believed that the samples where alkaloids and would be promising as leishmanicidal agents, and that fractionation would contribute to this activity. However, only the subfractions (SF5-6; dihydrocorynantheol, and SF-9), which were suggestive of alkaloids, displayed activity against *L. (L.) amazonensis*, whereas the FA was inactive. These results suggest alkaloids isolation is important to antipromastigote activity. Indeed, many alkaloids isolated from plants showed leishmanicidal properties^18^.

Some studies describe natural compounds as capable of promoting morphological changes in parasites^19,20^. The alkaloid β-carboline-1-propionic acid, can induce apoptosis^21^ and it can induce great ultrastructural damage in *L. amazonensis* promastigotes, including some evidence of apoptosis, such as vacuolization of the cytoplasm, presence of myelin-like figures, and swollen kDNA networks^22^. Another alkaloid, voacamine, induced intense cytoplasm disorganization, presence of autophagic vacuoles, changes in the kinetoplast, mitochondrial membrane, and mitochondrial ridges in promastigotes^19^.

Ultrastructural analysis (SEM and TEM) of promastigote forms treated with FrDCL, SF5-6, SF8, and SF9, displayed great morphological changes, which occurred in a concentration-dependent fashion. Despite the fact that FrDCL presented a moderate antipromastigote effect, based on studies developed by members of this research group, and due to the presence of metabolites other than alkaloids in this fraction, we decided to include it in this study to check whether the combination of molecules could promote a different set of morphological changes upon the parasites, than the isolated alkaloids.

In this sense, the morphological analysis revealed that parasites treated with FrDCL presented changes in shape, in the flagellar pocket, and in the cytoplasm. The reduction in concentration contributed to damage reduction, causing dilation of the flagellar pocket and alteration in the flagellar membrane. The FrDCL fraction externalization by the parasite through vesicles resulting from the evagination of the flagellar pocket membrane, suggests that this sample, perhaps, could inhibits hydrolytic^23,24^ or proteolytic enzymes^25^ and thus be responsible for the damage to the parasite. However, further investigations are needed to elucidate the possible mechanism of action.

Studies reported that endocytosed material is internalized from the flagellar pocket, and then vesicles sprout out from the pocket membrane. These vesicles appear to fuse and discharge their content into intracellular organelles comparable to the endosomes of mammalian cells^23,24^. There is little information about the factors that control access to the flagellar pocket, and about the physical and biochemical properties of constituents of the pocket lumen. The presence of certain hydrolases (eg, acid phosphatase) within the lumen of the flagellar pocket is an indication that the pocket may act as a prelysosomal hydrolytic compartment^23,24^.

When considering the study about microorganism proteases, it is important to keep in mind that these enzymes play important roles in the physiology of these organisms, as well as in the pathologies caused by them^26^. Among them, cysteine proteases are the most investigated proteolytic enzymes in *Leishmania*. These enzymes play important roles in *Leishmania,* such as virulence, viability maintenance, parasite morphology, invasion of the host's mononuclear phagocytic system, and the modulation of its immune response^27,28^, thus constituting attractive targets for chemotherapy for the treatment of leishmaniasis.

Studies carried out the purification and biochemical characterization of three serine proteases from *Leishmania (L.) amazonensis* promastigotes called LSP^29^, restricted mainly to intracellular compartments^30^, as megasomes and to the flagellar pocket^31^.

Protease activity can be regulated in cells or organisms in different ways, including protease inhibition through specific inhibitors^32^. These inhibitors are valuable tools for investigating the biochemical properties and biological functions of proteases^33^ and the inhibition of these enzymes by specific inhibitors can be an important strategy in the production of potent antimicrobial agents^34^.

When analyzed by TEM, the *L. (L.) amazonensis* promastigotes treated with 10−5 M of the protease inhibitor ShPI-I, obtained from the sea anemone *Stichodactyla helianthus*, presented flagellar pocket with vesicles inside, in addition to bubble formations in the membrane of its pocket, a structure of intense exocytic/endocytic activities. In the cytoplasm, the presence of vesicles that resemble autophagic vacuoles was observed, and this would a be result of intense exocytic/endocytic activity induced by this inhibitor. Moreover, all parasites exhibited changes in shape^25^. These changes were not seen in control cells. Thus, other classic protease inhibitors such as N-tosyl-L-phenylalanine chloromethyl ketone (TPCK) and benzamidine (Bza) were tested in this study and they also caused changes in the flagellar pocket. In another study, it was observed that ultrastructural abnormalities in the flagellar pocket and lysosome/endosome were seen exclusively with cysteine protease inhibitors regardless of their chemical composition, for example vinyl sulfone *versus* dihydrazide. This observation suggests that the cellular alterations seen are due specifically to inhibition of the cysteine proteases of *Leishmania*^27^.

We highlight that changes in the flagellar pocket and parasites shape were also observed in the present study. However, whether these enzymes participate in the exocytosis/endocytosis pathway through the processing of intracellular proteins or even in the morphological maintenance of *Leishmania* remains to be elucidated^25^.

Similar to FrDCL, treatment with a lower concentration of SF5-6 promoted changes in the shape of the flagellar pocket, which presented vesicles with granular material. Increasing the concentration of treatments increased the intensity and occurrence of damage.

The promastigotes treated with FrDCL and SF5-6 presented lipid inclusions. The biogenesis of lipid bodies is involved in cellular homeostasis in eukaryotes, as well as during infection by intracellular pathogens^35^. Given the role in cellular homeostasis, the accumulation of lipid bodies observed in the treated parasites may be a result of cellular disturbances and loss of homeostasis. Moreover, the intracellular presence of lipid bodies can indicate changes in the content of phospholipids and sterols^36^.

Similarly, changes in flagellar pocket were observed in parasites treated with dihydrocorynantheol. In addition, the retraction of the cell body of the parasites was observed, which is an alteration frequently detected in apoptotic cells^37^. During the initial stages of apoptosis, rounding and retraction of the cell is observed. This occurs due to constituent’s proteolysis of the cytoskeleton^38^, facts that were observed in the present study.

Among other changes caused by dihydrocorynantheol, there is the dilation of cisterns in the Golgi complex. This organelle is essential for the processing of lipids and proteins from the endoplasmic reticulum (ER)^39^. Like the endoplasmic reticulum, the Golgi complex can trigger the apoptotic process in response to stressful situations (excess of proteins unfolded from the endoplasmic reticulum, changes in membrane traffic or in its structure, among others). Some studies demonstrate that the Golgi complex not only suffers the consequences of the apoptotic process, but also plays an active role in the triggering of this type of cell death^40^. All of this reinforces the hypothesis that the leishmanicidal mechanism of dihydrocorynantheol should involve apoptosis.

In the treatment of promastigotes with the SF9, changes in shape, flagellar pocket, kinetoplast, presence of vacuoles, lipid inclusions and small projections of the parasite's cytoplasmic membrane were observed. Most changes are similar to the ones induced by other alkaloids and FrDCL. It is worth mentioning that treatment with SF9 induced the alteration in the kinetoplast of the parasites. This change can be a result of the fragility of the surrounding mitochondrial membrane. Thus, changes in the mitochondrial membrane could indirectly explain the changes observed in the kinetoplast^41^. The kinetoplast appears physically associated with the mitochondrial membrane and the basal body by thin filaments, which forms a complex structure known as the tripartite binding zone, which is essential for the positioning of the mitochondrial genome and its correct segregation during cell division^42^.

## Conclusion

In this study, alkaloids isolated from *A. nitidum* are promising as leishmanicidal and changes in flagellar pocket may be involved in this activity. Among alkaloids, the most promising is dihydrocorynantheol and, perhaps, apoptosis is involved in this activity. However, further studies need to be carried out to confirm the occurrence of this process in these parasites, since specific markers for apoptosis were not used in this investigation.

## Methods

### Plant material

The bark of A. nitidum, locally known as Caranapauba were collected in Santa Bárbara do Pará, PA-Brazil (S 01° 10 ′946’ W 048° 11 ′715’) during May 2013. The plant material was identified by Dra. Rafaela Trindade and the exsiccate was incorporated into the MG Herbarium of the Museu Paraense Emílio Goeldi, under no. MG206608. In the present study we used a wild plant collected from a virgin forest of the Amazon, and our work posed no risk of extinction for the species. During the collection, we took all care to remove the barks so as not to cause any damage to the species, in addition, only a small proportion of the barks were collected. The species were kept integrated and survived the collection. The project complies with national and international guidelines and legislation and is registered on the platform of the national management and Genetic Heritage System and Associated Traditional Knowledge (SISGEN), whose provided license to collect the species under registration A92C186.

### Phytochemical studies

The trunk barks of *A. nitidum* were dried in an oven (40 °C for 7 days; Medicate Medical Products, model MD 1.2), grounded, and subjected to maceration (7 days) to obtain the EE, which was subjected to an acid–base partition, resulting in the neutral (FN) and alkaloid fractions (FA)^43^. The EE was also subjected to extraction under reflux, yielding the following fractions: hexane (FrHEX), dichloromethane (FrDCL), ethyl acetate (FrAcOEt) and methanol (FrMeOH)^44^.

**Figure 5:**
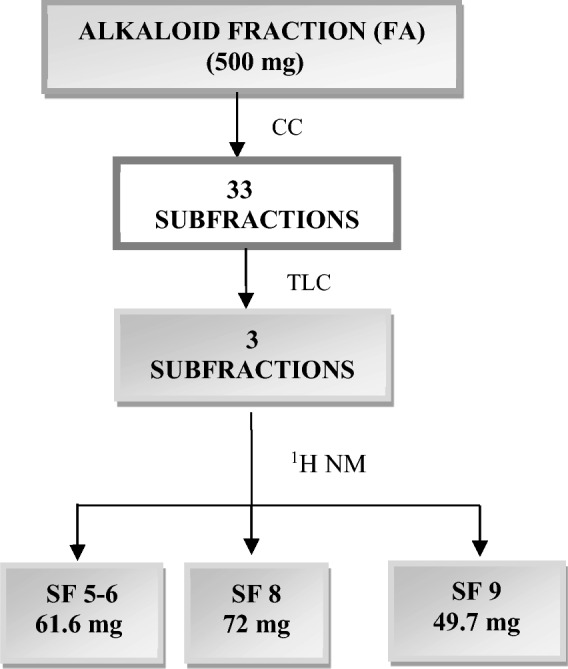
Flowchart for obtaining SF 5–6, SF8 and SF9 subfractions.

The FA (500 mg) was subjected to fractionation in an open silica gel chromatographic column (CC; 63–200 mm; Macherey–Nagel), using mobile phases of increasing polarity (dichloromethane, ethyl acetate e methanol; Quimis; Isofar) resulting in 33 subfractions which were analyzed in Thin Layer Chromatography (TLC) on silica gel (Macherey–Nagel) and combined, resulting in three subfractions to SF5-6, SF8 and SF9 (Fig. 5). Structural identification was performed by hydrogen nuclear magnetic resonance (^1^H NMR; Bruker Ascend 400) at 25ºC, using deuterated methanol (MeOD; Sigma-Aldrich®) to solubilize the samples. Thus, EE, fractions and subfractions were analyzed by High Performance Liquid Chromatography coupled to a Diodes Array Detector (Waters e2695 and Waters 2998).

The analysis was performed in a RP 18 column (45 × 250 mm, 5 µm), using a mixture of water acidified with 0.01% of trifluoroacetic acid (eluent A; Tédia) and acetonitrile acidified with 0.01% of trifluoroacetic acid (eluent B; Tédia) as the mobile phase. A linear elution gradient was used: 70–30% of eluent B for 15 min, 60–40% of eluent B for 20 min and 50–100% of eluent B for 25 min. The temperature was maintained above 26 °C, flow of 1 mL/min, UV detection at 220–400 nm^43^.

### Antipromastigote activity

The promastigote form of *Leishmania (L.) amazonensis* (MHOM/BR/2009/M26361) was grown in NNN medium (Novy-Nicolle-Mcneal) and later transferred to RPMI (Roswell Park Memorial Institute medium; Gibco) supplemented with 10% of denatured fetal bovine serum (Cultilab materials for cell culture), penicillin G 10,000 IU (Gibco)/mL and streptomycin 10,000 µg (Gibco)/mL and maintained at 25° C ± 1 °C through weekly passages. In the assay of antipromastigote activity, promastigotes were quantified in a Neubauer chamber (Improved) and then distributed in cell culture plates (Techno Plastic Products-TPP), previously pre-dosed with different concentrations of the samples and amphotericin B (positive control; Cristália pharmaceutical products). The plates were incubated and after 72 h of treatment, the tetrazolium salt (Sigma-Aldrich) was added followed by a new incubation. After 4 h of incubation, dimethyl sulfoxide (Dinâmica) was added and, then, the reading was performed in a microplate reader (Biotek, model ELX 808) at 490 nm. The samples were considered active when the IC_50_ ≤ 100 µg/mL, moderately active IC_50_ 101–200 µg/mL, inactive when the IC_50_ ≥ 200 µg/mL as adapted from^45^. The 50% Inhibitory Concentration (IC_50_) is the concentration that causes a 50% reduction in growing cells (viable) and was determined by the GraphPad Prism software.

### Morphological analysis

The parasites treated with the active samples were analyzed by SEM (LEO 1450 VP) and TEM (ZEISS model EM 906 e ZEISS model EM 900), according to the method described by^46^.

In SEM analysis, promastigotes (4 × 10^6^ parasites/500 µL) were distributed in 24-well plates (TPP) pre-dosed with FrDCL, SF5-6, SF8 and SF9 (50 µL/well), after that they were incubated 72 h/26 °C. Afterwards, the contents of each well were removed, centrifuged and then fixed in PHEM (PIPES—piperazine-n n'-bis(2-ethanesulfonic acid) 20 mM, EGTA (Ethylene glycol-bis(β-aminoethyl ether)-N,N,N′,N′-tetraacetic acid tetrasodium salt) 10 mM, MgCl_2_ (Magnesium chloride) 5 mM, KCl (Potassium chloride) 70 mM 1X) + Paraformaldehyde (PFA; Electron Microscopy Sciences) 4% + 2.5% glutaraldehyde in 0.1 M sodium cacodylate buffer (Electron Microscopy Sciences), pH 7.2 for 2 h. Then, the parasites were washed with 0.1 M sodium cacodylate buffer (pH 7.2) and deposited in glass coverslips with a solution of 0.1% poly-L-lysine (Sigma-Aldrich, St. Louis) for 2 h.

Post-fixation was performed with a solution containing 1% osmium tetroxide (Sigma-Aldrich, St. Louis) and potassium ferrocyanide (Electron Microscopy Sciences) for 1 h. Then, the coverslips were washed with 0.1 M sodium cacodylate buffer, pH 7.2, dehydrated in a graded ethanolic series (70, 80, 90 and 100%; Merck) and dried by the critical point method using CO_2_. The samples on the coverslips were fixed on an appropriate support and metallized with a platinum film for about 2 min. The parasites were analyzed by SEM (LEO 1450 VP).

In the TEM analysis, the parasites were centrifuged, and the pellet resuspended in a 1% glutaraldehyde solution with 4% paraformaldehyde in 0.1 M PHEM buffer (pH 7.4), and 2.5% sucrose for fixation for 1 h. After fixation, the cells were washed with 0.1 M sodium cacodylate buffer (pH 7.4) and post-fixed in a solution containing 1% osmium tetroxide and 1% potassium ferrocyanide for 1 h in the dark, washed with buffer 0.1 M cacodylate (pH 7.4), and then with distilled water and immersed in a contrasting 1% uranyl acetate (Electron Microscopy Sciences) solution in 25% acetone (MERCK) for 1 h. Then, samples were dehydrated in graded acetone (50, 70, 90 and 100%). Afterwards, the cells were slowly impregnated in increasing concentrations of Epon (Electron Microscopy Sciences) diluted in acetone until pure Epon (100%). The samples were then infiltrated with pure Epon + DMP-30 (2,4,6-Tris (dimethylaminomethyl) phenol) and left in a support for polymerization in an oven at 60 °C for 48 h. The blocks were cut in an ultramicrotome (Leica, model EMVC6) and ultrathin sections were contrasted with 5% uranyl acetate and, subsequently, with lead citrate for further analysis by TEM (ZEISS model EM 906 and ZEISS model EM 900).
